# One-step thoracotomy approach for atrial-esophageal fistula repair without cardiopulmonary bypass

**DOI:** 10.1007/s11748-023-01952-5

**Published:** 2023-06-17

**Authors:** June Lee, Jeashin Yoon, Seok Beom Hong, Yong Han Kim, Hwan Wook Kim, Do Yeon Kim

**Affiliations:** Department of Thoracic and Cardiovascular Surgery, Seoul St. Mary’s Hospital, College of Medicine, The Catholic University of Korea, 222 Banpo-daero, Seocho-gu, Seoul, 06591 Republic of Korea

**Keywords:** Atrial-esophageal fistula, Thoracotomy, Cardiopulmonary bypass

## Abstract

Atrial-esophageal fistula is an extremely rare disease and a life-threatening complication after catheter ablation for atrial fibrillation. There is no consensus on the management or repair for atrial-esophageal fistula which has a high mortality rate. Here, we describe a lateral thoracotomy approach focused on simplifying the repair procedure for atrial-esophageal fistula in two patients.

## Introduction

Catheter ablation for atrial fibrillation (AF) is widely utilized and considered safe (with 6% complications) [1]. Although atrial-esophageal fistula (AEF) is an uncommon disease, it is a life-threatening complication after catheter ablation for AF [2, 3]. The onset of AEF occurs several days to 2 months after catheter ablation. Its symptoms are diverse and include neurologic changes [2]. Because of its uncommonness, there is no gold standard for repairing AEF, which has a high mortality rate (up to 80%) [4]. Herein, we illustrate a lateral thoracotomy approach focusing on a simple repair for AEF in two patients.

## Technique

### Case 1

**Fig. 1:**
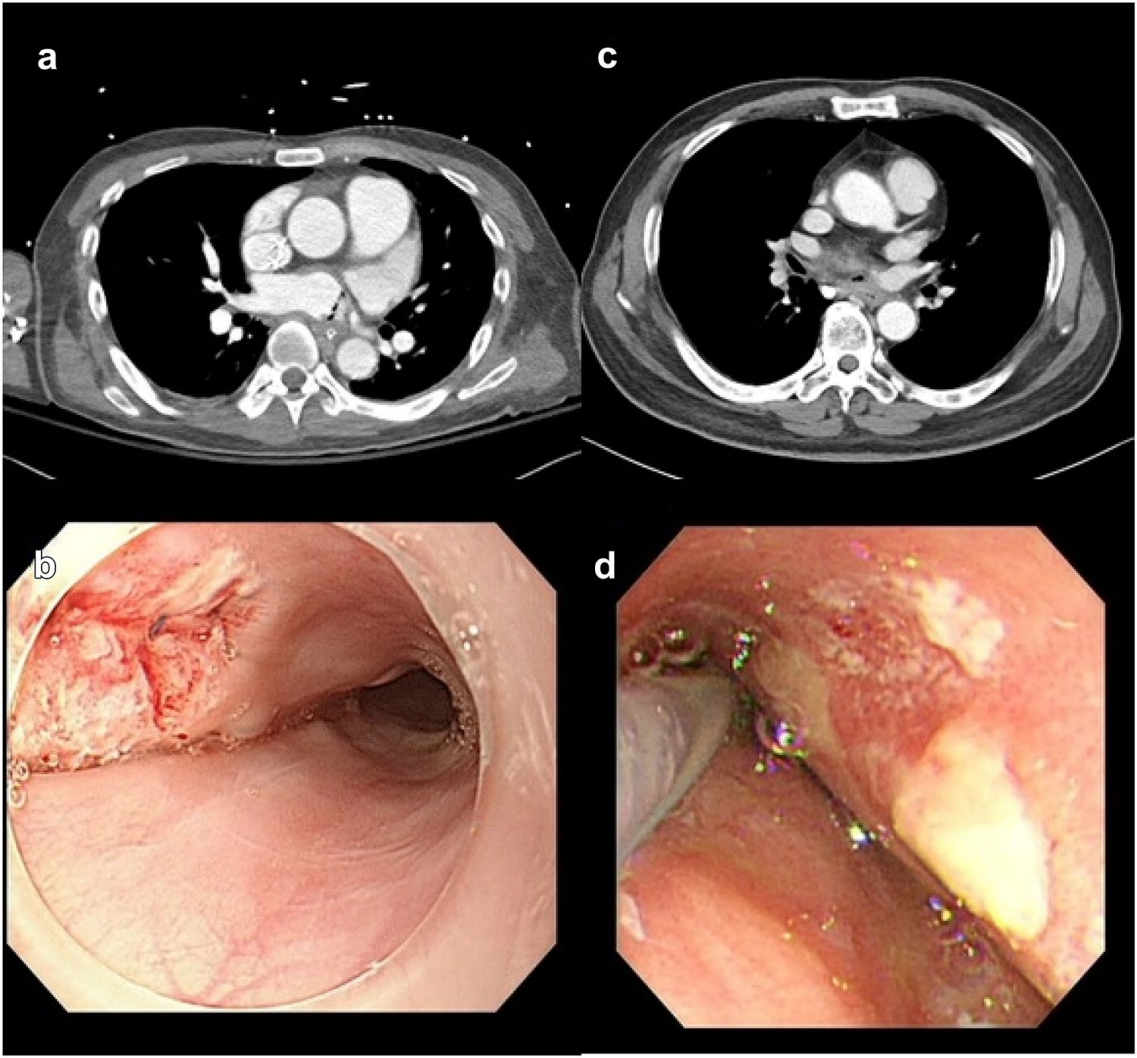
Perioperative findings about atrial-esophageal fistula in two patients. **a** A CT scan of the chest showing abnormal linear air densities in the left atrium demonstrating an AEF in case 1. **b** An endoscopic finding with a well-repaired previous fistula site in case 1. **c** A CT scan of the chest revealing air bubbles at the mediastinum suggestive of an AEF in case 2. **d** An endoscopic finding was unremarkable for a prior AEF site covered with a whitish exudate scar in case 2

A 61 year-old man presented to the emergency department with a history of 3 days of fever. He had a history of radiofrequency catheter ablation of AF 1 month prior to admission, end-stage renal disease requiring hemodialysis, and pacemaker insertion. After admission to the hospital for evaluating the cause of fever, altered mental status was noted. Brain magnetic resonance imaging (MRI) showed a multifocal embolic infarction. *Streptococcus salivarrius* and *Streptococcus mitis/oralis* were found in blood culture. Chest computed tomography (CT) demonstrated AEF (Fig. 1a).

**Fig. 2:**
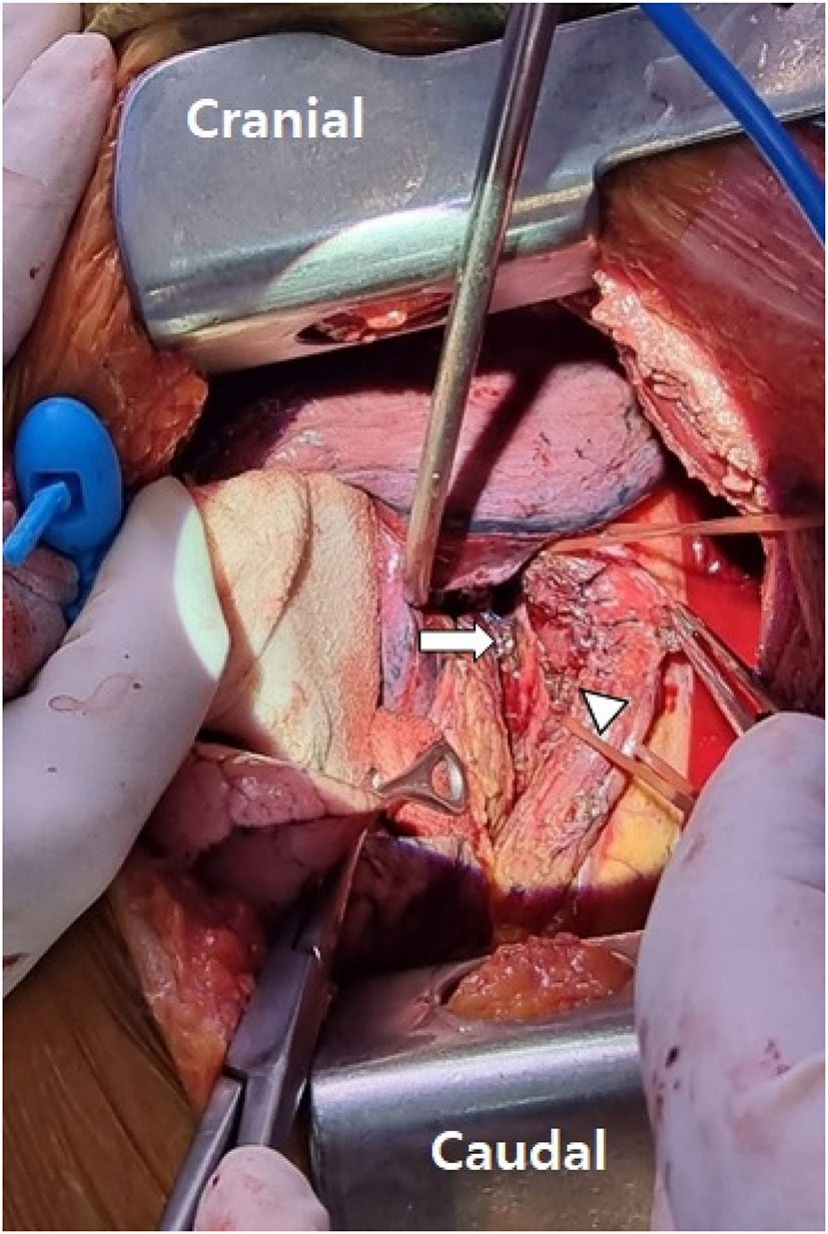
Intraoperative findings showing a well-exposed, ligated AEF site by the left thoracotomy approach in case 1. The white arrow indicates the pericardial side of AEF, and the white arrowhead indicates the esophageal side of AEF

A surgical strategy was urgently planned. After endotracheal general anesthesia, the patient was placed in the right lateral decubitus position. Single-lung ventilation was initiated. A thoracotomy was performed on the left 5th intercostal space. After left lung retraction, the pericardium was opened, revealing severe adhesions. Accessing the fistula through the pericardial space was attempted but was abandoned due to excessive adhesions. Attention was then turned to the outside of the pericardium. After retracting the left lung forward, we accessed below the left main bronchus and confirmed the left superior and inferior pulmonary veins. Then we approached behind the pulmonary veins and performed dissection. After opening the mediastinal pleura on the left hilum, the esophagus behind the pericardium was identified. Efforts were made to avoid any vascular damage because it is located more medially than the descending aorta. Between the esophagus and the posterior pericardial wall, the fistula site was observed (Fig. 2). We confirmed the 3 mm fistula with esophageal traction cautiously. Since there were no CT findings or surgical evidence suggesting an abscess, we judged that the situation was not severe in terms of localized infection. From posterior pericardial reflection, three pledgeted 4–0 prolene sutures were gently placed on the fistula on the pericardial side. Fistula ligation and resection were followed by an additional primary repair of the esophageal defect. The mucosal defect was repaired with interrupted 4–0 vicryl sutures, and the muscularis was reapproximated with interrupted 3–0 silk sutures. The repair procedure was finished after considerable irrigation of the chest and a chest tube was inserted.

Postoperatively, the patient was treated for sequelae from the initial stroke and pneumonia. After 1 week without oral nutrition, we confirmed a well-repaired fistula site without residual lesions on endoscopy (Fig. 1b). The patient was discharged on postoperative day 31 with a tracheal tube.

### Case 2

A 70 year-old man underwent radiofrequency catheter ablation for AF. Fifty days following the procedure, he was admitted for cognitive decline. Multiple cerebral infarct lesions were observed on brain MRI. Blood cultures were taken to determine the cause of the patient’s fever. Multiple *Streptococcus* spp. were confirmed. A CT scan of the chest was suggestive of an AEF (Fig. 1c).

Surgery was urgently performed. The surgical approach was the same as in patient 1. After left thoracotomy on the 5th intercostal space, inspection of the pericardial space showed calcified adhesions. Through the posterior mediastinal space, the esophagus was exposed and pulled gently. The 3 mm fistula on the posterior aspect of the pericardium was isolated and carefully ligated with several pledgeted 4–0 prolene sutures. After fistulectomy, primary esophageal repair was performed in the same manner as the first case. After irrigation, a chest tube was then inserted. The wound was closed in a layered fashion.

The patient's postoperative program was focused on respiratory care with tracheostomy and rehabilitation for the cerebral infarction sequelae. Endoscopic findings were nonspecific after 1 week of fasting (Fig. 1d). The patient was discharged on postoperative day 23 with a tracheal tube.

## Comments

Early diagnosis and surgical intervention are important in AEF. There are several treatment options for AEF, including esophageal stenting, intracardiac repair, extracardiac repair, and esophageal repair [2, 3, 5, 6].

Intracardiac repair of AEF requires sternotomy and cardiopulmonary bypass. A two-stage approach using intracardiac repair and esophageal repair has several disadvantages. It requires repositioning of the patient [7]. Another hybrid technique [8], including intracardiac repair and endoscopic clipping, has a risk for failure of the clipping procedure and the need for thoracic operation in cases of refisulization.

Patients with single-step repairs of AEF using a thoracotomy approach, with or without cardiopulmonary bypass, have also been reported [9, 10]. Our patients had a treatment strategy similar to the method reported by Khandhar et al. [10]. However, we did not use intercostal muscle flaps or any stapling devices. Unlike what was described by Khandhar and others, we focused on repairing the fistula in the simplest way possible.

The single-step lateral thoracotomy for AEF has several advantages in patients who are deemed to be free of left atrial active bleeding to the pericardial space. This lateral thoracotomy method can reduce operating time and eliminate the need for a cardiopulmonary bypass and a surgical repositioning. An AEF can be visually and reliably removed in one step. Also, due to the approach through the thoracic cavity, it has the advantage of effectively removing localized abscesses around the esophagus. Even if the problem on the left atrium side remains, there is room for future open heart surgery. Since stent insertion using endoscopy is not employed, the risk of air embolism associated with endoscopy can be eliminated. The lower esophagus runs toward the left hemithorax and passes behind the posterior wall of the left atrium, the left thoracotomy approach allows for better exposure of the lower esophagus. However, it is essential to exercise extreme caution during esophagus and fistula manipulation to prevent the occurrence of air embolism. Similar to our two cases who are less likely to have active bleeding of the left atrial wall, we can verify the intrathoracic findings first and close the fistula. Even in patients with multiple cerebral infarcts, it becomes burdensome to use cardiopulmonary bypass, since there is concern about cerebral hemorrhagic changes.

In conclusion, our experience suggests that one-step repair for AEF via lateral thoracotomy without cardiopulmonary bypass might be feasible in selected patients, especially in those with concomitant cerebral infarction.

## Data Availability

The data that support the findings of this study are available from the corresponding author, DYK, upon reasonable request.
